# Surgical site infections in Vietnamese hospitals: incidence, pathogens and risk factors

**DOI:** 10.1186/1753-6561-5-S6-O54

**Published:** 2011-06-29

**Authors:** NV Hung, TA Thu, NQ Anh, NN Quang, AK Lennox, S Salmon, D Pittet, LM McLaws

**Affiliations:** 1 Infection Control Department, Bach Mai Hosptial, Vietnam; 2 Department of Science and Training, Ministry of Health, Ha Noi, Vietnam; 3 Division of Infectious Diseases, College of Medicine, University of Florida, Florida, USA; 4 School of Public Health & Community Medicine, University of New South Wales, Kensington, NSW, Australia; 5 Infection Control Programme, University of Geneva Hospitals and Faculty of Medicine, Geneva, Switzerland

## Introduction / objectives

Globally surgical site infections (SSIs) are associated with substantial morbidity, mortality and imposed the financial burden to hospitals, patient families and societies. This study is to determine the incidence, aetiology, and risk factors associated with SSIs in Vietnam.

## Methods

During 2009, a 3-month prospective survey was carried out in seven hospitals included national and provincial facilities across Vietnam. SSIs were diagnosed according to the criteria established by the Centers for Disease Control and Prevention, USA. All patients who underwent a surgical procedure and were inpatients in trauma, general surgery and obstetrics wards were enrolled in the study. The aggregated data included patient demographics, medical and surgical information, microbiological parameters, and SSI categories.

## Results

During the study period, 4,413 patients underwent a surgical procedure. The overall crude SSI incidence was 5.5%. Risk factors independently associated with SSIs were as follows: age≥ 30 yrs (adjusted odds ratio [aOR]: 1.9; 95% confidence interval [CI]: 1.3 – 2.9), clean-contaminated wound (aOR: 1.7; CI: 1.2 - 2.8), contaminated wound (aOR: 1.8; CI: 1.1 - 3.2), dirty wound (aOR: 3.2; CI: 1.8 -5.7), duration of surgery > 120 minutes (aOR: 1.9; CI: 1.3 - 3.4), or small bowel surgery (aOR: 4.0; CI: 2.1 - 7.6). *Escherichia coli* (38.7%) and *Klebsiella pneumonia* (16.1%) were two most commonly identified pathogen associated with SSI.

## Conclusion

Our findings indicate that SSIs constitute a major problem in Vietnamese hospitals. These data suggest areas for intervention and implementation of SSI prevention policies.

## Disclosure of interest

None declared.

